# Analysis of Older Adults in Spanish Care Facilities, Risk of Falling and Daily Activity Using Xiaomi Mi Band 2

**DOI:** 10.3390/s21103341

**Published:** 2021-05-11

**Authors:** María del Carmen Miranda-Duro, Laura Nieto-Riveiro, Patricia Concheiro-Moscoso, Betania Groba, Thais Pousada, Nereida Canosa, Javier Pereira

**Affiliations:** 1 CITIC (Centre for Information and Communications Technology Research), TALIONIS Group, Elviña Campus, University of A Coruna, 15071 A Coruña, Spain; carmen.miranda@udc.es (M.d.C.M.-D.); patricia.concheiro@udc.es (P.C.-M.); b.groba@udc.es (B.G.); thais.pousada.garcia@udc.es (T.P.); nereida.canosa@udc.es (N.C.); javier.pereira@udc.es (J.P.); 2 Department of Physiotherapy, Medicine and Biomedical Sciences, Faculty of Health Sciences, Oza Campus, University of A Coruna, 15071 A Coruña, Spain; 3 Department of Health Sciences, Faculty of Health Sciences, Oza Campus, University of A Coruna, 15071 A Coruña, Spain

**Keywords:** daily steps, falls, health-related quality of life, nursing home, occupational therapy, physical activity, remote monitoring, sleep, wearable technology, wristband

## Abstract

Background: Presently the use of technological devices such as wearable devices has emerged. Physical activity monitoring with wearable sensors is an easy and non-intrusive approach to encourage preventive care for older adults. It may be useful to follow a continuous assessment of the risk of falling. The objective is to explore the relationship between the daily activity measured by Xiaomi Mi Band 2 and the risk of falling of older adults residing in or attending care facilities. Methods: A cross-sectional study was conducted on three different institutions located in Galicia (autonomous community) (Spain). Results: A total of 31 older adults were included in the study, with a mean age of 84 ± 8.71 years old. The main findings obtained were that a greater number of steps and distance could be related to a lower probability of falling, of dependency in basic activities of daily living, or of mobility problems. Conclusions: The importance of focusing on daily steps, intrinsically related to the objective assessment of daily physical activity, is that it is a modifiable factor that impacts different aspects of health and quality of life.

## 1. Introduction

Currently, it is well documented that aging populations are progressively increasing. Older people represent 20.3% of the European population over 65 years old, after Japan with 28% [1,2]. Also, life expectancy increases, which means that people are expected to live longer, increasing the risk of chronic diseases and higher levels of dependency in daily life [3]. Therefore, as people age, they are more likely to need third-party support, which complicates the choice of aging in place and independently [4,5]. In this case, especially in Spain, there are three different options: living at home with home assistance either by a professional or by a nonprofessional carer, living at home and attending a day center, or going to a long-term care facility [6].

In Spain, and particularly in the Galicia autonomous community, older people represent 25.5% of the population, and the ratio of residential vacancies to 100 older people is around 3:1 [7]. The estimated number of older people living in Spain’s nursing homes is 81.4% [8]. Usually, an older person who is institutionalized loses capacity and skills, which affects independence in daily life, presenting an imbalance in their occupational performance [9]. Likewise, they are more likely to have poor quality of life, low cognitive and physical status, affecting their daily lives [9].

During the aging process, chronic diseases can derive from a decreased physical activity level [10,11]. Over time, some studies have concluded that higher daily physical activity levels reduce chronic diseases in all age groups [12]. Thus, the World Health Organization (WHO) recommended adults aged 65 years get between 150 and 300 min of moderate-intensive physical activity per week [13].

The most inactive and active people in aging populations are at the highest risk of falls [14]. Thus, one of the most frequent syndromes in the older population is the risk of falling, which is the second leading cause of death worldwide and considered to be one of the most common reasons for a decreased level of physical activity and for institutionalizing older adults [14]. For this reason, falls are identified as an urgent global health challenge that, despite the pandemic situation, is gaining great importance, as well as indicating the importance of being physically active [15]. The incidence of falls in community-dwelling older people over 65 years is around 30%, and in residential care facilities, approximately 50% [14].

The risk of falling is associated with gait and balance problems, which also impact physical activity levels. Agmon et al. [16] demonstrated that the increased gait variability was a strong predictor of falls in older adults. Gait problems mean losing independence, a significant reduction in quality of life, increased risk of falling, and mortality and morbidity [16]. Some of the main fall factors are age, previous falls, living alone, use of medicines, gender, medical conditions (i.e., circulatory disease, depression, arthritis), impaired mobility and gait, sedentary behavior, fear of falling, nutritional deficiencies, impaired cognition, visual impairments, hearing loss, and foot problems [14].

People with a recent history of falls can acquire a fear of falling [14]. Occupational therapists have a key role in addressing the risk and fear of falling because it may limit a person’s ability to engage in a meaningful occupation and lead to a sedentary lifestyle. As Wu et al. mentioned [16], occupational therapists may collaborate to manage the person’s concerns about falling to maintain daily functions and independence [16]. Under these circumstances, new strategies and tools should be sought to assess aspects related to the risk of falling from an occupational perspective, for example, technological tool use. Furthermore, in a previous scoping review about the use of technology by occupational therapists in dealing with falls, there was not found any experience with wearable devices [17].

The existing literature is widely related to human activity detection for daily activities. It includes the detection of falls, the analysis of objective gait, the analysis of different signs and symptoms associated with neurological disorders (i.e., Parkinson’s disease), among others [18]. Regarding fall detection, the solutions are focused on visual sensors, telecare solutions with user interaction, mobile applications, or wearable systems [19]. Wearables constitute any mobile device worn on the body, called on-body sensors, such as inertial measurement units, smart watches, wristbands, or Holter electrocardiogram monitors [20]. They provide objective and quantitative measures from controlled and unsupervised environments. As mentioned, they allow the development of accurate treatment plans and disease monitoring [21]. These objective techniques are body-fixed motion sensors, ranging from switches, pedometers, actometers, goniometers, accelerometers, and gyroscopes, mainly for physical activity assessment. Mechanical pedometers, known as step counters, are the basic wearable sensors to measure human motion. They are often used to compare data from the wristbands [22,23].

The analysis of using wearable technology has been developed in unsupervised environments under free-living conditions instead of in laboratories or clinical visits [20]. Wearable devices can track nearly everything, from providing early stroke detection to monitoring physiological parameters, quantifying physical activity, monitoring sleep quality, determining gait structures, and measuring plantar pressures and shear [24]. Also, the key advantage of wearable sensors is that no dedicated laboratory environment is needed to extract parameters of interest. Thus, mobility assessments, such as gait and balance tests, can be performed in any clinical setting and during a routine clinic visit [25]. In addition, monitoring people in a daily living environment and over continuous periods may become more feasible and ecological [20,26,27].

While using conventional geriatric assessment tools or questionnaires provides information on the purpose of the activity, wearable devices can quantify the motion performed. Both provide complementary information to the researchers, which are not interchangeable [28,29]. Likewise, as supported by Yang et al. [30], studies in fall risk assessment should recommend using wearable technologies to supplement nursing home assessment tools. In fact, at present, the use of technological devices such as wearable devices has been emerging. For instance, wearable sensors are an easy and non-intrusive approach to encourage preventive care for older adults. It may be useful to continuously monitor and assess daily activities in real-life environments [31].

Literature on the study topic has referred to the main wearable devices used in older adults’ populations: wristbands, activity monitors, or accelerometers. The main objectives of using these devices are: (1) to explore the relationship between sleep behavior and gait performance [16], (2) to validate the devices by step count [32], (3) to evaluate the feasibility and efficacy of the device [33,34], (4) to determine the validity of a device compared to Actigraph [35,36], or (5) to understand the use of wristbands by older adults conducting qualitative or mixed studies [37,38,39,40,41].

The trusted wearable device with a research purpose is the Actigraph accelerometer (ActiLife, Pensacola, E.E.U.U.) [34]. Notwithstanding, Paul et al. determined that this accelerometer undercounts steps in older adults, especially in people with mobility impairments [32,35]. Furthermore, despite it being a budget option, DeGroote et al. [42]’s study reported a great agreement between the data from Actigraph and the Xiaomi Mi Band (Xiaomi, Beijing, China), which makes it a competent low-cost choice.

O’Brien et al. [33] supported that wristband activity trackers are an accepted method for recording daily physical activity among older adults. Previous studies have encouraged the use of the Xiaomi Mi Band, reporting that it seems to be one of the best packages for its price [43]. Also, Puri et al. [39]’s study showed that the Xiaomi Mi Band has an 80% acceptance by older adults, more than the Microsoft Band (50%), (Microsoft, E.E.U.U.). In this line, previous studies such as that of Mičková et al. [44], have used the Xiaomi Mi Band to assess physical activity, steps, and self-reported health in a group of older adults [44].

Tudor-Locke et al. [45] found that being physically active is associated with higher levels of functional health, lower risk of falling, and improved cognitive health.

Accordingly, this study aims to examine the association between Xiaomi Mi Band 2 parameters, especially for daily steps, with older adults who are at risk of falling, residing, or attending a care facility. This study aims to describe their fall history and determine the influence of daily steps and sleep on dependency in basic activities of daily living (B.A.D.L.), having a cognitive impairment, and having any problem associated with health-related quality of life (HRQoL). Finally, this study aims to analyze the utility of the Xiaomi Mi Band 2 to assess older adults’ daily activity and sleep.

## 2. Materials and Methods

### 2.1. Study Design

An observational descriptive cross-sectional study was conducted on three different institutions located in Galicia autonomous community (Spain), all of them providing services as a residence and day center. It is part of the GeriaTIC project [46] (Clinical Trials NCT03504813), in which two institutions participated, and another project (Clinical Trials NCT04592796), in which one institution participated. The three institutions have similar characteristics and similar populations. The study began in March 2017, and it was completed in December 2019.

### 2.2. Participants

A purposive sampling was used to recruit the participants from the three institutions. They were eligible for study entry if they met the following criteria:

- aged over 65 years old;
- resided in or attended a nursing home or day center;
- able to walk 3 m;
- able to provide written informed consent;
- understood, spoke, and read Spanish proficiently;
- not having requested a transfer to another center;
- agreed to wear the wristband for 30 days (during the day and night).

### 2.3. Data Collection

The participants had to wear the Xiaomi Mi Band for 30 days, 24 h a day. The assessment tools selected were administrated the first day of wristband use to draw a baseline about the sample. A data set about the variables was compiled [47].

### 2.4. Tools and Measures

#### 2.4.1. Institution Database

The variables selected were sex (male or female), age, care facility (nursing home or day center), marital status (widow or not), body mass index (normal weight or overweight), and the number of diagnoses from the medical record.

The diagnoses most closely related to the risk of falling were selected and classified into the following groups:

- Diagnoses of any of these physical conditions: osteoporosis, osteoarthritis, dizziness and giddiness, rheumatoid arthritis, abnormalities of gait and mobility, or multiple sclerosis.
- Diagnoses of any of these cognitive conditions: Alzheimer’s disease, dementia, or age-associated cognitive decline.
- Diagnoses of other health conditions: hypertension, visual impairment, diabetes, or hearing loss.

Other variables included were the number of certain assistive aids used (glasses, hearing aid, or anti-decubitus pillow), the number of mobility aids (cane or walker), the number of falls in the last 12 months, and fall classification profile (non-fallers with any fall in the last 12 months, or faller with one or more falls in the last 12 months) [30].

In addition, the institution database obtained the most recent Barthel Index Score, Tinetti Index Score, and the presence or not of cognitive impairment. Barthel Index measures the level of dependency in B.A.D.L. such as feeding, bathing, grooming, dressing, bowel control, bladder control, toilet use, transfers, mobility on level surfaces, and up and downstairs. In the Barthel Index the score ranges between 0 and 100 points, 100 is considered independency in B.A.D.L., and >100 is considered any level of dependency in B.A.D.L. [48]. The Tinetti Scale assesses the risk of falling based on gait and balance, and the total score is between 0 and 28, considering ≥24 as no risk of falling and <24 as the risk of falling [49].

#### 2.4.2. Health-Related Quality of Life

The EuroQol-5D-5L (EQ-5D-5L) was used to explore the perception of each participant about their HRQoL. It evaluates four elements [50]. The first element consists of a descriptive system of five dimensions: mobility (walking ability), self-care (washing or dressing), usual activities (i.e., work, study, household chores, family activities, or leisure time activities), pain/discomfort, and anxiety/depression; these are assessed as: (1) no problems, (2) slight problems, (3) moderate problems, (4) severe problems, or (5) extreme problems/inability. In this case, we considered those having any problem or no problem to analyze [50]. The second element was a Visual Analog Scale (VAS), in which the person rates his/her perceived health from 0 (the worst imaginable health) to 100 (the best imaginable health) [50]. Finally, the third and fourth elements (the EQ-5D-5L Index and the Severity Index, respectively) are two indexes calculated from the descriptive system’s scores. The EQ-5D-5L Severity Index score between 0 (absence of problems) to 100 (more severity), and the EQ-5D-5L Index between 0 (state of health similar to death) and 1 (better health status) [50].

#### 2.4.3. Xiaomi Mi Band 2

The participants were monitored for 30 days with the Xiaomi Mi Band 2 located on their dominant hand. This band has a battery of about 30 days, so it was unnecessary to load during the study period. This device was used to count steps in order to provide a means of objectively quantifying total daily activity. Individual Gmail accounts were created for each participant to collect the data from the wristbands, and through the Mi Fit mobile application, the data were extracted to an Excel document. The parameters obtained were the 30-day averages based on the following variables:

- Daily activity: analyzed through the number of daily steps and the daily distance covered by each participant in meters. Based on Tudor-Locke’s study, <3000 steps indicated a low level of physical activity and 3000–10,000 steps indicated a moderate physical activity level [23].
- Sleep: analyzed in minutes in four different parameters (daily deep sleep, daily shallow sleep, total daily sleep, and awake time in bed during the night). The Sleep Foundation recommendation was used to reference older adults’ adequate sleep of around 7–8 h, which corresponds to 420–480 min per day [51].

Data from the wristbands were compared by the institution’s professionals, and the researchers asked the participants weekly about their sleep and daily activity to ensure that the data represented their daily lives. Likewise, if the Mi Fit mobile application showed null values, this meant that the person had not worn the wristband or even that the device was failing. In this study, the institution’s professionals supervised if the participant wore the wristband daily.

### 2.5. Statistical Analysis

Statistical analyses were conducted using International Business Machines Corporation (I.B.M.^®^) Statistical Package for the Social Sciences (S.P.S.S.^®^) version 25. First, descriptive analysis was done using the data as means and standard deviation or frequencies and percentages, as appropriate.

Concerning inferential analysis, in the case of two categorical variables, the chi square test was implemented, contrasted with the Fisher test, since more than 20% of the boxes had values lower than 5. For these reasons, the categorical variables were grouped into two different values to create 2 × 2 contingency tables. The effect size was checked with Cramer’s V, which has values between 0 and 1, so values close to 1 indicated a strong association.

On the other hand, the normality of the numerical variables was analyzed with the Shapiro–Wilk test, which is intended for small samples. Variables with a normal distribution (Shapiro–Wilk test with *p* > 0.05) were analyzed with Student’s T test (tested in each categorical variable) and Pearson Correlation (correlation with other numerical variables), and those with abnormal distribution (Shapiro–Wilk test with *p* < 0.05) were analyzed with the Spearman correlation test (correlation with other numerical variables) and Mann–Whitney U test (numerical variables with abnormal distribution and categorical variables). Spearman and Pearson coefficient “rho” (values from 0 to 1) determined the correlation’s magnitude, and for Mann–Whitney U test results, GPower 3.1. software was used to calculate the effect size (the estimated magnitude of the relationship) and the statistical power (the probability that the null hypothesis was accepted when the alternative hypothesis was true) of the correlations and associations according to Hedges’ g, which was indicated with “g” and the statistical power indicated with “β”. Then g values below 0.2 indicated a small effect size, 0.5 of medium magnitude, and 0.8 indicated a high magnitude effect.

Regarding the significant associations previously explored, associations of risk of falling and Xiaomi Mi Band 2 parameters were analyzed using simple binary regressions, which were implemented to determine Odds Ratios (ORs) since the dependent variables are categorical and take two values (0, 1). The values of Cox and Snell R^2^ and Nagelkerke R^2^ indicate the percentage by which the regression explains the relationship between the variables. When the value is greater than 0.4, 40% of the regression explains the association between the variables. It can be considered a good result.

Lastly, the authors decided to apply a generalized model developed with Gamma distribution and logarithm link. This type of model is used for variables that do not have a normal distribution and have an integer and positive values, as in daily steps [52].

### 2.6. Ethical Concerns

The present study followed the ethical concerns of the Declaration of Helsinki for human research ethics. Moreover, participant confidentiality was ensured under Regulation 2016/679 and repealing Directive 95/46/E.C. of the European parliament to protect personal data. Participants received complete verbal and written information about the characteristics of the study and the implications derived from their participation in it. Once it was ensured that all participants fully understood the information provided, they agreed to participate in the study through the Informed Consent Document. The data of the participants were collected and preserved until the end of the study in coded mode.

## 3. Results

A total of 31 older adults were included in the study. As reported in Table 1, the mean age was about 84 years old. There was also homogeneity in gender, in which women represented 51.6% of the sample. Members of this sample mainly resided in a nursing home (n = 28) and were widows (83.9%), overweight (71%), had problems associated with any physical condition (54.8%) (i.e., osteoporosis, osteoarthritis, dizziness, and giddiness, rheumatoid arthritis, abnormalities of gait and mobility, or multiple sclerosis), with the presence of cognitive impairment (64.35%), with any level of dependency in B.A.D.L. (77.4%), took fewer than 3000 steps per day (87.1%), slept less than 420–480 min per day (54.83%), and used mobility aids (41.9%). Likewise, during the study, if any participant suffered a fall, this variable was not analyzed.

### 3.1. History of Falls

**Figure 1:**
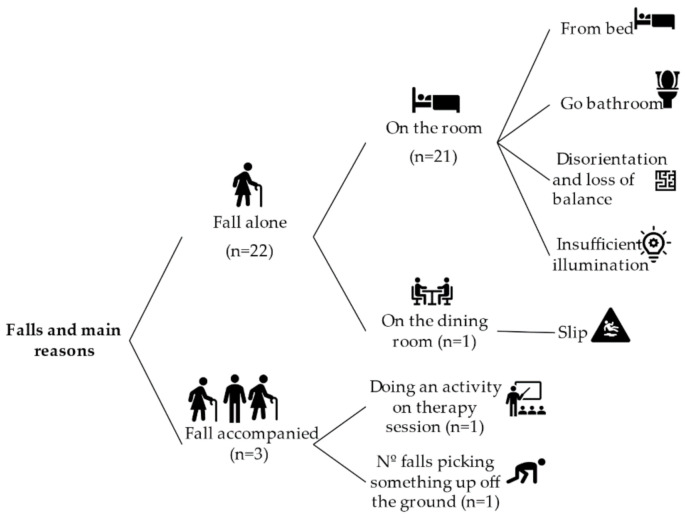
Fall distribution and main causes.

The history of falls expresses the number of falls experienced in the last 12 months before the present study. Figure 1 reports the distribution of falls and their main reasons. According to this, 45.2% of the sample (n = 14) had falls in the last 12 months. The total amount of falls was 25, and 32% of them had not identified the reason in the institutional database. Most of the falls happened when the person was alone (n = 22) and when he or she was in a room of the care institute (n = 21).

An analysis of the possible association between having a previous fall and the risk of falling was implemented. However, there was not found a significant association (*p* > 0.05). It should be noted that 11 of the 15 participants who had one or more previous falls were at risk of falling.

### 3.2. Risk of Falling

We investigated the risk of falling. We determined that the risk of falling may be associated with having any level of dependency in B.A.D.L. (*p* = 0.002, V = 0.618), taking fewer than 3000 steps per day (*p* = 0.007, V = 0.618), having four or more diagnoses (*p* = 0.006, V = 0.539), using mobility aids (*p* = 0.006, V = 0.532), having any physical condition (*p* = 0.018, V = 0.483), and using a walker (*p* = 0.03, V = 0.441).

Regarding the problems identified in the five dimensions of the EQ-5D-5L descriptive system, we found a positive and moderate association with mobility problems (*p* = 0.015, V = 0.585). However, there were identified negative strong associations with problems in self-care (*p* = 0.002, V = −0.483) and moderate association with problems in usual activities (*p* = 0.018, V = –0.483). No association between risk of falling and pain and discomfort or anxiety or depression was determined.

Moreover, we found associations between risk of falling and the number of diagnoses (F = 8, *p* = 0.007), the number of mobility aids used (F = 10, *p* = 0.003), the Barthel Index Score (F = 16, *p* > 0.001), the EQ-5D-5L Severity Index (F = 5.3, *p* = 0.028), and the EQ-5D-5L Index (F = 5.3, *p* = 0.028).

### 3.3. Health-Related Quality of Life

Related to HRQoL, the sample represented, as reported in Table 2, an EQ-5D-5L VAS of about 69 ± 15, EQ-5D-5L Index of about 0.68 ± 0.25, EQ-5D-5L Severity Index of about 22 ± 18, and a score greater than 50 in EQ-5D-5L VAS in 87.1% of the participants.

**Figure 2:**
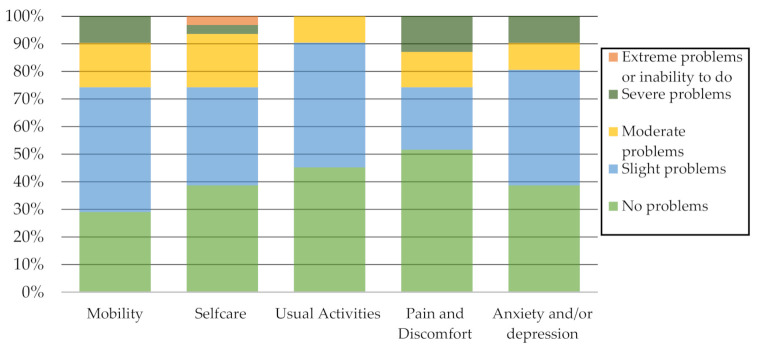
EQ-5D-5L Descriptive system dimensions.

Figure 2 reports the percentage of problems identified by the participants on each EQ-5D-5L descriptive system dimension. It should be noted that not identifying problems or identifying slight problems in mobility, self-care, usual activities, pain and discomfort, and anxiety and/or depression predominated.

### 3.4. Xiaomi Mi Band 2 Parameter Associations

The Student’s T test and Mann–Whitney U test were used to explore the association between the parameters measured by the Xiaomi Mi Band 2, which were the independent variables (daily steps, daily distance covered, daily deep sleep, daily shallow sleep, and daily awake time at night), with being or not being at risk of falling, the presence or not of any level of dependency in B.A.D.L., and the presence or not of cognitive impairment, which were the dependent variables (see Table 3).

With the risk of falling, there were strong associations of daily steps (*p* < 0.001, F = 27), daily distance covered (*p* < 0.001, F = 31), and weak association with daily awake time at night (*p* = 0.013, F = 0.127).

The strongest associations with dependency in B.A.D.L. were with daily steps (*p* < 0.001, g = −2.086, β = 0.99), daily distance covered (*p* = 0.005, g = −2.666, β = 0.99) and daily deep sleep (*p* = 0.005, g = −0.793, β = 0.54). However, no significant associations were found with cognitive impairment.

Related to the EQ-5D-5L descriptive dimensions, which were the independent variables, (see Table 4), strong associations were found with daily steps and mobility (*p* = 0.001, g = −1.432, β = 0.97), self-care (*p* > 0.001, g = 1.404, β = 0.97), and usual activities (*p* < 0.001, g = 0.956, β = 0.76), and a moderate association with pain and discomfort (*p* = 0.008, g = 0.860, β = 0.68), as well as similar associations were found with daily distance covered.

It was concluded that with a higher number of daily steps and daily distance covered, fewer problems were identified in mobility and more problems were identified with pain and discomfort. Moreover, we found a moderate association between daily shallow sleep and mobility (*p* = 0.005, F = 3.78), and with pain and discomfort (*p* = 0.003, g = 1.066, β = 0.84). Daily total sleep was associated with usual activities (*p* = 0.042, g = 0.654, β = 0.53) and a low association with pain and discomfort (*p* = 0.02, g = −0.071, β = 0.07). No associations were encountered with daily deep and daily awake time with the dimensions of the EQ-5D-5L descriptive system.

### 3.5. Correlations and Associations with Daily Steps

Daily steps had no relation with age, gender, and body mass index (*p* > 0.05). However, after the implementation of the Pearson Correlation (normal distribution) and Spearman Correlation (abnormal distribution), we identified other correlations. This analysis determined that the significant, strongest, and positive correlations with daily steps were with Barthel Index Score (*p* < 0.001, rho = 0.691), EQ-5D-5L Index Score (*p* < 0.001, rho = 0.603), and EQ-5D-5L VAS (*p* = 0.013, rho = 0.377). The negative strongest correlations with daily steps were the number of mobility life aids used (*p* < 0.001, rho = −0.625), EQ-5D-5L Severity Index Score (*p* < 0.001, rho = −0.564, β = 0.99), daily awake time at night (*p* < 0.01, rho = −0.506), number of diagnoses (*p* < 0.01, rho = −0.462), number of assistive aids (*p* < 0.05, rho = −0.367). We did not find correlation with age (*p* = 0.155, rho = −0.262) and Tinetti Score (*p* > 0.050, rho = −0.063).

### 3.6. Risk of Falling Binary Regression

Concerning the risk of falling, a binary regression model was carried out, obtaining a Cox and Snell R^2^ of 0.408 and a Nagelkerke R^2^ of 0.571, which could be considered a good fit model. The following variables were removed from the model because they did not have a predictive effect on the risk of falling: taking fewer than 3000 steps per day, physical conditions, mobility aids, problems in self-care, and problems in mobility. Thus, the final model suggested that a person at risk of falling was 24 times more likely to present any level of dependence (*p* = 0.016, OR = 24) and 11 times more likely to have four or more diagnoses (*p* = 0.038; OR = 11).

### 3.7. Xiaomi Mi Band 2 Parameters Binary Regressions

Binary regressions (see Table 5) were performed according to the significant associations reported in Table 3 and Table 4.

Regarding the binary regressions, higher level of daily steps had an association with more probabilities of not having a risk of falling (*p* < 0.05, OR = 1.004, R^2^ = 0.570–0.796), perceiving no problems in EQ-5D-5L Mobility (*p* < 0.01, OR = 1.085, R^2^ = 0.411–0.587), perceiving no problems in EQ-5D-5L Usual activities (*p* < 0.05, OR = 0.999, R^2^ = 0.261–0.349), not having a dependency in B.A.D.L. (*p* < 0.05, OR = 0.913, R^2^ = 0.375–0.571), and having a minor probability of not perceiving problems in EQ-5D-5L Self-care (*p* < 0.05, OR = 0.998, R^2^ = 0.424–0.576).

Related to the other Xiaomi Mi Band 2 parameters we observed that greater daily distance covered was associated with greater probability of not having a risk of falling (*p* < 0.05, OR = 1.006, R^2^ = 0.534–0.746), greater probability of not having a level of dependency in B.A.D.L. (*p* < 0.05, OR = 1.002, R^2^ = 0.388–0.591), greater probability of not having perceived problems in EQ-5D-5L Mobility (*p* < 0.01, OR = 1.002, R^2^ = 0.420–0.599) and having a minor probability of not having problems in EQ-5D-5L Self-care (*p* < 0.05, OR = 0.998, R^2^ = 0.453–0.615). Daily awake time at night was associated with a minor probability of not having a risk of falling (*p* < 0.05, OR = 0.913, R^2^ = 0.346–0.483). Moreover, daily shallow sleep was associated with not having perceived problems in EQ-5D-5L Mobility (*p* < 0.05, OR = 1.024, R^2^ = 0.334–0.477) and a minor probability of not having perceived problems in EQ-5D-5L Pain and Discomfort (*p* < 0.05, OR = 0.986, R^2^ = 0.303–0.404).

### 3.8. Generalized Model of Daily Steps

A generalized model was applied with a distribution of Gamma and Logarithm link function. The dependent variable was daily steps with the stronger correlations and associations that were previously found.

Results from the generalized model are reported in Table 6. Many of the variables with significant association or correlation were removed due to not being significant predictors. Finally, we obtained that daily steps had a negative association with the risk of falling (*p* < 0.001, OR = 0.312) and when a person had any level of dependence in basic daily activities (*p* = 0.27, OR = 0.567). It meant that a person had 0.312 times higher risk of falling or 0.567 times higher dependency on basic activities when taking fewer steps per day, which meant that the more steps you take each day, the more likely you are to not be at risk of falling or becoming dependent; specifically, a person is about twice likely to not be at risk of falling or becoming dependent if he/she has an adequate level of daily steps.

**Figure 3:**
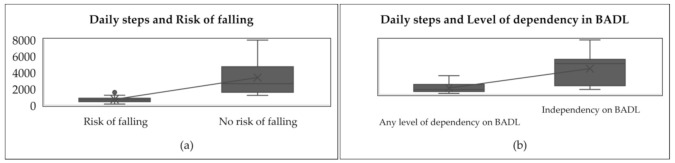
Associations between daily steps and level of dependency in B.A.D.L. and risk of falling. (**a**) it is shown the comparison of daily steps between people with risk of falling and people with no risk of falling. (**b**) it is shown the comparison of daily steps between dependent people and independent people.

The associations between daily steps and the level of dependency in B.A.D.L. and risk of falling are shown in Figure 3.

Figure 3 shows that people at risk of falling took fewer steps per day (between 146 and 1920) in comparison to people with no risk of falling, who took more steps per day (between 1188 and 7942). In other words, the average number of steps taken by the people with a risk of falling was lower (937 ± 738), while those taken by people with no risk of falling was greater (3755 ± 2527). Likewise, dependent people took fewer steps per day (between 146 and 2739) compared to independent people, who took more steps per day (between 729 and 7942). By comparing both in Figure 3, it is shown that dependent people’s steps mean per day was lower (937 ± 738) while independent people’s steps mean per day was greater (3755 ± 2527).

## 4. Discussion

The present study examined the Xiaomi Mi Band 2 parameters and older adults’ health related to physical activity and risk of falling. The main findings obtained were that a greater number of steps and distance could suppose a lower probability of presenting a risk of falling, dependency in B.A.D.L., or perception of mobility problems. In this way, as Patterson’s study [53] concluded, there is no agreement on what dose of physical activity should be performed to maintain a person’s functional independence. However, it is known that with moderate physical activity levels, there can be significant results [53]. Likewise, the relationship between staying physically active and engaging in regular physical activity, with health benefits, particularly in fall rate reduction has been well documented for decades [54].

The number of steps taken by the participants who did not perceive mobility problems, were not at a risk of falling, and were independent in B.A.D.L. were between 2503 median steps in a range of 7256, a mean of 3366 steps ± 2139, and a median of 4509 in a range of 7213, respectively. In other words, considering these three aspects, the number of daily steps ranged from 2500 to 6000 steps approximately. Similar data were obtained in the O’Brien study, in which the intermediate steps of older adults were 2500–4000 [33]. However, according to Tudor-Locke et al. [45], this range fits a sedentary profile. These authors suggested that below 6000 daily steps could not provide health benefits [45]. It suggests that physical activity levels should be increased, although it should be noted that the mean age of the participants was 84 ± 8.71 years old, with 87.1% of the participants who took fewer than 3000 steps per day. Thus, it is not clear whether this level of physical activity may significantly affect their health for this population.

Daily steps are a modifiable factor intrinsically related to the objective assessment of daily physical activity. They have a strong impact on health in any population, but especially in older adults. It affects their level of independence and quality of life, taking into account the repercussions of falls [55]. This study suggested that wearable devices, like Xiaomi Mi Band 2, may be used for appropriate assessments, which can help to identify those with an increased risk of falls to reduce the negative impact of falls in older adults [56]. A total of 45.2% of the sample had previous falls, considering that their normal incidence in nursing homes is 50% [14]. Despite the pandemic situation, falls are still gaining great importance [15], making them a focus of attention. In this case, health professionals and caregivers played a central role in mitigating unnecessary risk-taking [57].

The association between having a previous fall and the risk of falling was investigated. Nevertheless, no association was found. However, previous studies have indicated that older fallers have a high prevalence of fall risk factors and are at risk of functional decline [58]. As aforementioned, it was possible to observe a lower risk of falling at a higher level of physical activity.

Regarding sleep, in this study, we observed that daily awake time at night was weakly associated with the risk of falling (*p* = 0.013, F = 0.127). Although the data were not supported by strong associations and knowing that it is necessary to consider the quality of sleep as measured by deep sleep, shallow sleep, and total sleep, they showed an important aspect of using wearables devices. Wearable devices continuously monitor the person, which provides the approximate time that the person has been awake at night, and, therefore, they can help to understand their needs.

In this way, Stefan et al. [59] reported that older adults with short sleep duration are less likely to meet physical activity guidelines. In contrast, those who report long sleep duration and good sleep quality are more likely to meet physical activity guidelines [59]. In the present study, the participants who perceived problems with mobility were likely to have more daily shallow sleep. In contrast, people with perceived pain and discomfort problems had a higher risk of getting less daily shallow sleep. In other words, we found that people with subjective pain and discomfort may get less shallow sleep. However, it should be compared with deep sleep to examine the quality of sleep, but in this case, no significant associations were found to conclude. Similarly, no significant relations were found with cognitive impairment, and perceived problems with usual activities, or total sleep parameters and deep sleep.

The existing literature has supported a relationship between short sleep duration and injury from falling [54]. In addition, maintaining daily routines was associated with a reduced rate of insomnia in older adults [60]. In the present study, 54.83% of the participants slept less than 420–480 min, which is the adequate range of sleep per day [51], while participants with a risk of falling slept 360 ± 118 min per day in comparison with those with no risk of falling, who slept 421 ± 85 min per day. It means that people who are not at risk of falling, sleep more, and have sleep levels that are within the appropriate range, although it was not possible to conclude a significant relationship.

Regarding the risk of falling, we found that a person at risk of falling is 24 times more likely to present any level of dependence and 11 times more likely to have four or more diagnoses following the risk factors collected by the WHO, who specified different medical conditions, mobility and gait impaired, and sedentary behavior, among others [14]. Thus, the risk of falling may be related to dependency and comorbidity. The association with the use of mobility aids has not been established, which may affect the number of daily steps. Also, it has not been possible to determine whether these aids can be a facilitator or a barrier in older adults’ daily lives.

### 4.1. Limitations

The main limitations identified are the size and heterogeneity of the sample, since they influenced our being able to draw strong conclusions or associations between some aspects of physical activity and the risk of falling. Other risk factors for falls, such as fear of falling, medication, nutrition, or environmental adaptation, should be considered.

### 4.2. Further Work in the Field

It would be important to carry out a case-control study with an appropriate sample size to determine whether having a risk of falling influences the number of daily steps taken, as this study suggests that people who are not at risk of falling could take more steps per day. Also, further studies should include other variables such as fear of falling, medication, nutrition, or environmental adaptation, to explore how they influence the risk of falling.

### 4.3. Clinical Implications

Wearable devices can make older people aware of their physical activity and sleep. As Hopman reported, physical activity promotion is a difficult challenge to the habits of older adults [61]. In this way, as mentioned above, occupational therapists have a key role in promoting healthy routines and habits. In addition, any health professional and caregiver play a central role in mitigating unnecessary risk-taking [57]. Thus, an interdisciplinary team is necessary to address the risk of falling. Carers should receive the necessary training from occupational therapists or other health providers to prevent or reduce falls.

Wristbands may be an effective and fast way to evaluate people without requiring extended time for professionals to determine their day-to-day needs. It will now be useful in the COVID situation to observe how this situation has affected people’s physical activity and sleep levels.

## 5. Conclusions

The main findings obtained were that a greater number of steps and distance could be related to a lower probability of presenting a risk of falling, dependency in B.A.D.L., or perception of mobility problems.

Based on the results, cognitive impairment does not have strong associations with any of the Xiaomi Mi Band 2 parameters selected (steps, distance, deep sleep, shallows sleep, total sleep, and awake time at night).

Regarding sleep, the results suggest that people at risk of falling tend to be awake longer at night, independent people get more deep sleep, people who identify problems in their usual activities have a lower total sleep time, and finally, people who identify pain or discomfort have less light sleep and sleep in total.

The risk of falling may be related to dependency and comorbidity. However, we cannot determine whether mobility aids can be a facilitator or a barrier in older adults’ daily lives.

Lastly, wearable devices continuously monitor the person, which can help to understand their needs. The importance of focusing on daily steps, intrinsically related to the objective assessment of daily physical activity, is because it is a modifiable factor that impacts different aspects of health, quality of life, and risk of falling.

## Figures and Tables

**Table 1:** Sample main characteristics.

Characteristic	N (%)/Mean (±SD)
Age	84 (±8.71)
Women	16 (51.6%)
Residing in a nursing home	28 (90.3%)
Widow	28 (83.9%)
Overweight	22 (71%)
Problems of physical conditions	17 (54.8%)
Cognitive impairment	20 (64.5%)
Dependency in basic daily activities	24 (77.4%)
Risk of falling	21 (67.7%)
Take fewer than 3000 daily steps	27 (87.1%)
Sleep less than 420–480 daily minutes	17 (54.83%)
Use of mobility aids	13 (41.9%)
EQ-5D-5L VAS > 50	26 (12.9%)

**Table 2:** EQ-5D-5L scores.

	EQ-5D-5L VAS	EQ-5D-5L Index	EQ-5D-5L Severity Index
Mean	69	0.68	22
Standard deviation	±15	±0.25	±18
Minimum	40	0.03	0
Maximum	100	1	65

**Table 3:** Xiaomi Mi Band 2 parameter associations.

Dependent Variables	Independent Variables					
	Daily Steps	Daily Distance	Daily Deep Sleep	Daily Shallow Sleep	Daily Total Sleep	Daily Awake Time at Night
Risk of falling	^a^ **	^a^ **	^a^	^a^	^a^	^a^ *
Yes	720 (±480)	446 (±61)	150 (±67)	271 (±93)	360 (±118)	56 (±44)
No	3366 (±2139)	2161 (±477)	174 (±20)	303 (±83)	421 (±85)	19 (±11)
Level of dependency in B.A.D.L.	^b^ ***	^b^ **	^b^ **	^a^	^a^	^a^
Yes	696 (2593)	449 (1651)	164 (180)	285 (385)	360 (533)	151 (17)
No	4509 (7213)	3008 (4817)	180 (149)	303 (252)	446 (141)	17 (41)
Cognitive impairment	^a^	^a^	^b^	^b^	^b^	^a^
Yes	1915 (±2048)	1232 (±1381)	174 (264)	308 (278)	390 (209)	46 (±42)
No	953 (±834)	569 (±507)	102 (186)	261 (385)	363 (533)	42 (±39)

^a^ Student’s T test shown with Mean (Standard Deviation). ^b^ Mann–Whitney U Test shown Median (Range). *p*-value > 0.05 *, *p*-value > 0.01 **, *p*-value > 0.001 ***. The effect size was in contrast with Hedges’ g.

**Table 4:** Associations between descriptive dimensions of EQ-5D-5L and Xiaomi Mi Band 2 parameters.

Dependent Variables	Independent Variables					
	Daily Steps	Daily Distance Covered	Daily Deep Sleep	Daily Shallow Sleep	Daily Total Sleep	Daily Awake Time at Night
Mobility	^b^ **	^b^ **	^a^	^a^ **	^a^	^b^
Any problem	683 (4363)	435 (2927)	147 (±68)	255 (±91)	354 (±117)	27 (151)
No problem	2503 (7256)	1654 (4849)	183 (±57)	345 (±43)	440 (±67)	22 (102)
Self-care	^b^ ***	^b^ ***	^a^	^a^	^a^	^b^
Any Problem	2339 (7256)	1543 (4849)	174 (±59)	317 (±61)	424 (±67)	19 (105)
No problem	519 (1774)	316 (980)	147 (±70)	258 (±98)	351 (±125)	27 (151)
Usual activities	^a^ *	^a^ *	^b^	^b^	^b^ *	^a^
Any Problem	2435 (±2095)	1534 (±1442)	169 (247)	308 (224)	420 (307)	159 (±71)
No problem	864 (±1036)	558 (±691)	173 (226)	252 (385)	360 (533)	157 (±64)
Pain and discomfort	^b^ **	^b^ **	^b^	^b^ **	^b^ *	^a^
Any problem	1263 (7500)	799 (5021)	176 (264)	326 (215)	420 (294)	43 (±41)
No problem	661 (1774)	433 (914)	160 (209)	248 (385)	360 (533)	46 (±41)
Anxiety and/or depression	^b^	^b^	^a^	^b^	^a^	^b^
Any problem	830 (4834)	513 (3256)	159 (±59)	312 (346)	389 (±120)	24 (121)
No problem	751 (7796)	462 (5224)	157 (±72)	281 (282)	378 (±108)	27 (150)

^a^ Student T test shown with “Mean (Standard Deviation)”. ^b^ Mann–Whitney U Test shown in the boxes with “Median (Range)”. *p*-value > 0.05 *, *p*-value > 0.01 **, *p*-value > 0.001 ***. The effect size was in contrast with Hedges’ g.

**Table 5:** Binary regression of Xiaomi Mi Band parameters.

Dependent Variable	Independent Variable	OR 95% CI	Cox and Snell R^2^	Nagelkerke R^2^
No risk of falling	Daily steps	1.004 (1.001–1.008) *	0.570	0.796
	Daily distance covered	1.006 (1.001–1.011) *	0.534	0.746
	Daily awake time at night	0.913 (0.843–0.989) *	0.346	0.483
Independency on B.A.D.L.	Daily steps	1.001 (1.000–1.003) *	0.375	0.571
	Distance daily covered	1.002 (1.000–1.004) *	0.388	0.591
	Daily deep sleep	1.014 (0.995–1.033)	0.124	0.189
EQ-5D-5L Mobility dimension–no problems	Daily steps	1.085 (0.926–1.272) **	0.411	0.587
	Daily distance covered	1.002 (1.001–1.004) **	0.420	0.599
	Daily shallow sleep	1.024 (1.003–1.045) *	0.334	0.477
EQ-5D-5L Self-care dimension–no problems	Daily steps	0.998 (0.996–1.000) *	0.424	0.576
	Daily distance covered	1.088 (0.811–1.460) *	0.453	0.615
EQ-5D-5L Usual activities dimension–no problems	Daily steps	0.999 (0.998–1.000) *	0.261	0.349
	Daily distance covered	0.999 (0.997–1.000)	0.233	0.312
	Daily total sleep	0.994 (0.985–1.003)	0.106	0.142
EQ-5D-5L Pain and Discomfort dimension–no problems	Daily steps	0.999 (0.997–1.000)	0.321	0.428
	Daily distance covered	0.998 (0.995–1.000)	0.352	0.469
	Daily shallow sleep	0.986 (0.974–0.999) *	0.303	0.404
	Daily total sleep	0.993 (0.983–1.003)	0.225	0.300

OR = Odds Ratio, CI = confidence interval. Adjusted by sex, age, and body mass index. * *p* < 0.05; ** *p* < 0.01.

**Table 6:** Generalized model of daily steps.

Predictor	OR 95% CI
Risk of falling	0.312 (0.161–0.568) ***
Any level of dependence on basic daily activities	0.567 (0.281–1.150) *

* *p* < 0.05; *** *p* < 0.001.

## Data Availability

A data set was published in Mendeley Data [46].
